# Visual field testing for glaucoma – a practical guide

**Published:** 2012

**Authors:** David C Broadway

**Affiliations:** Consultant ophthalmic surgeon, Department of Ophthalmology, Norfolk & Norwich University Hospital, and Honorary Reader, University of East Anglia, Norwich, UK.

**Figure:**
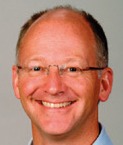
David C Broadway

## Part 1 Understanding visual field testing

Examining visual fields is an integral part of a full ophthalmic evaluation. Several methods for assessing visual field loss are available, and the choice of which to use depends on the patient's age, health, visual acuity, ability to concentrate, and socio-economic status. Available techniques can test the full field (including confrontation, tangent screen, Goldmann perimetry and automated perimetry), or assess just the central field of vision, such as the Amsler Grid (Figure 1).

If undertaking or interpreting visual field tests, it is a good idea to undergo the tests yourself. You will probably find that undergoing a visual field test can be difficult, requiring an understanding of what is required, an ability to perform tasks quickly, and high levels of concentration. Some methods require patients to press a buzzer when they see a target, and this requires quick reflexes and nimble hands. Undergoing the experience yourself will enable you to see how it is possible for patients to be slow and to make mistakes; this will help you to be patient and realise that you may need to explain the test several times. It will also give you greater insight when interpreting the test results, and can be very useful for teaching others how to explain the process to patients.

This article focuses on some of the more practical aspects of visual field testing, with an emphasis on assessing glaucoma.

### Key facts about visual field testing

1. Manual and/or automated visual field testing is subjective: it is totally dependent on the co-operation and responses of the patient. Poor results that are difficult to interpret are often due to the fact that the patient either did not understand what was required, or did understand but was unable to respond.
2. Abnormalities in the visual field are a sign of damage anywhere in the visual system from the retina through to the brain's visual cortex. Visual field defects are, therefore, not limited to glaucoma. It is very important to examine the retina and optic disc carefully to assess whether or not a visual field defect matches the appearance of the disc and retina, or fits with other clinical signs. One should be very wary of the person with extensive field loss, which seems genuine, where examination of the retina and optic disc are normal. T This person may have a neurological condition (e.g. a brain tumour) or theymay have had a stroke and not have glaucoma at all.

**Figure 1:**
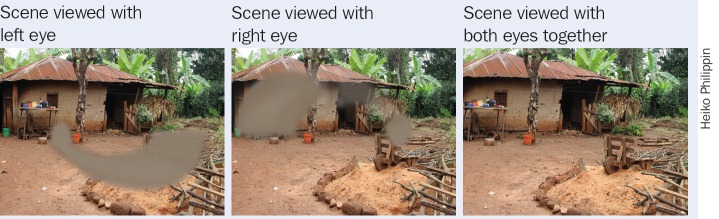
Asymmetrical visual field loss in glaucoma can lead to late presentation as with both eyes open the patient sees no defect
  These images represent what a scene may look like to someone with different visual field defects in each eye. The left eye has inferior field loss, and the right eye has superior field loss. Because the defects do not overlap, the field defects will not be apparent when the scene is viewed with both eyes together.
3. The combination of good visual acuities with full visual fields leads to excellent functional vision and both are very important. Loss of visual acuity can be very disabling, but so too can extensive loss of peripheral visual field. Loss of visual field, particularly the lower field, makes walking around difficult and slow, and people can easily lose confidence. Even in advanced glaucoma, when only a small area of peripheral visual field remains, health care workers should do all they can to preserve this remnant of vision, which can be very important for the patient's independence and dignity.
4. For diagnostic purposes, it is important to test each eye separately. This is because non-congruous defects in each eye (e.g. a superior defect in the visual field of the right eye and an inferior defect in the left eye) could be missed when testing both eyes together as the normal areas of field in one eye overlap the defects in the other eye. This leads to a normal binocular field of vision when both eyes are open (see Figure 1). This is clearly a good situation as far as the patient is concerned, but means that further extensive loss of field can occur without the person being aware of it. So, test the field of vision in each eye separately – at every visit!
5. Early glaucomatous visual field defects are subtle and easily missed. Even with modern automated and sensitive visual field analysers, glaucomatous visual field loss is not evident until at least 30% of the retinal ganglion cell axons that make up the optic nerve have been lost. Progression of visual field loss in untreated glaucoma can be quite slow, and signs of deteriorating disease can therefore be missed quite easily.
6. With all forms of visual field testing, record the patient's name or identification number, eye tested, visual acuity, the date, pupil size, whether the pupil was dilated, whether the upper lid had to be taped up, and/or if any corrective lenses were used. In addition, comment on patient co-operation, fixation, and perceived reliability in performing the test.
7. There are two major types of perimetry. Kinetic perimetry involves the detection of moving targets and static perimetry involves the detection of a stationary target. Static testing in general is superior to kinetic perimetry in detecting slopes and scotomata (field defects), and tends to be more reliable and consistent, particularly for detecting glaucomatous visual field loss.
8. In order to detect glaucomatous field loss, it is important to test for differences in the superior and inferior hemi-fields (above or below the horizontal raphé) and hunt for defects such as a nasal step, which is typical of glaucoma.
9. In order to detect neurological field loss, test for differences either side of the vertical meridian.

### New technologies and the future

Other technologies are available and being developed in the hope that formal visual field testing will become easier, more reliable, more affordable, and more widespread – using equipment that can detect glaucoma earlier than standard perimetry. These emerging technologies include:

- short-wavelength (blue–yellow) automated perimetry (SWAP)
- frequency doubling technology (FDT) perimetry
- motion displacement perimetry (MDP).

Over the next few years, it is highly likely that there will be a wider range of affordable visual field testing equipment available. This is good news since it means that glaucoma detection as well as management can be greatly improved. Training in visual field testing should be an integral part of ophthalmology residency programmes, as well as of training programmes for mid-level eye-care workers, so that awareness and expertise in glaucoma care become an integral part of the work of eye care workers everywhere.

Careful detection of visual field defects can be diagnostic of many ophthalmic and/or neurological conditions, including glaucoma. In glaucoma – as well as other conditions – it is vital to repeat visual field testing to track any changes over time.

Ideally, the same method of testing should be used for baseline and subsequent follow-up. In glaucoma, if visual field loss is progressive, it may mean that control of intraocular pressure (IOP) is inadequate. Be aware, though, that patients may remember to use their eye drops just before they come to the clinic, so that their IOP may appear to be controlled. It is far better to monitor control by assessment of the optic disc and visual fields than to rely on IOP alone.

**Figure 2:**
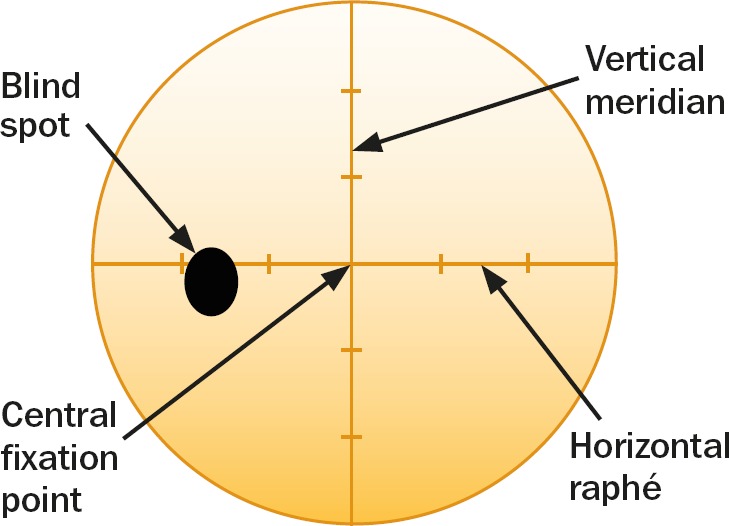
A normal visual field (left eye)

## Part 2 Features of glaucomatous visual field defects

Visual field loss can be diffuse (as with cataract or corneal opacification), but more commonly there are isolated defects. The visual field defects associated with glaucoma appear to be fairly non-specific, although typical loss fits with the arrangement of the retinal ganglion cell axons within the retinal nerve fibre layer of the retina.

**Figure 3:**
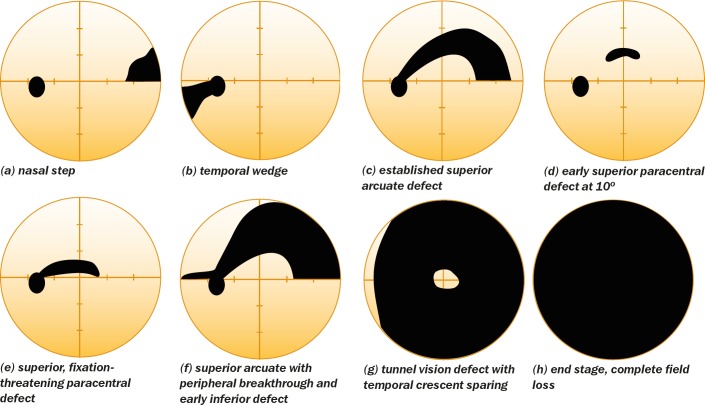
Glaucomatous field defects in left eyes

Relatively specific glaucomatous visual field defects (see Figure 3 for examples) include:

1. a nasal step defect obeying the horizontal meridian
2. a temporal wedge defect
3. the classic arcuate defect, which is a comma-shaped extension of the blind spot
4. a paracentral defect 10–20° from the blind spot
5. an arcuate defect with peripheral breakthrough
6. generalised constriction (tunnel vision)
7. temporal-sparing severe visual field loss
8. total loss of field.

The differential diagnosis for visual field loss similar to that seen with glaucoma includes:

- optic nerve head drusen
- retrobulbar optic neuritis
- tilted optic nerve
- anterior ischaemic optic neuropathy
- neurological field defects (especially bitemporal homonymous hemianopia)
- other rare optic nerve disorders
- focal retinal disease
- visual field artefacts.

**Figure 4:**
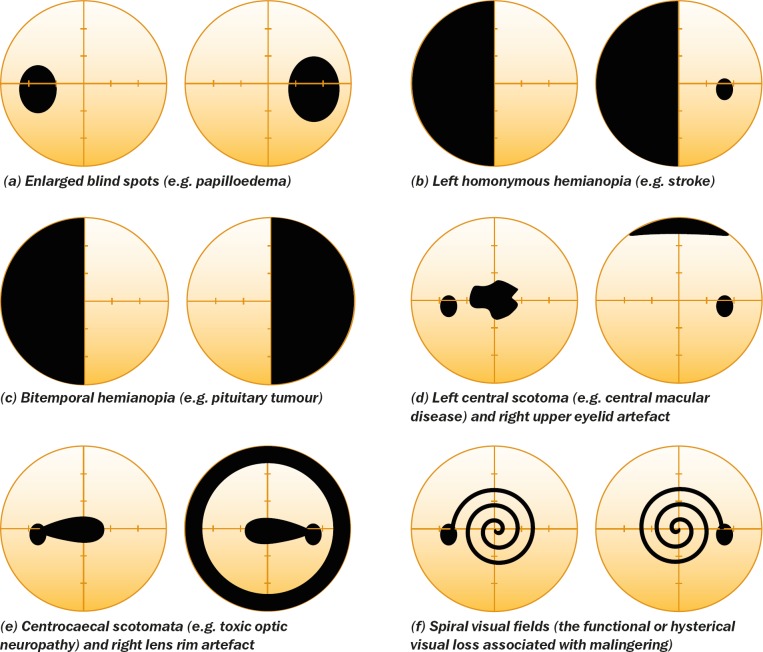
Non-glaucomatous bilateral visual field defects

Some non-glaucomatous visual field defects are shown in Figure 4.

## Part 3 Confrontation testing

The first thing you should do is to ask if the patient has noticed a visual field defect. The problem with this approach alone is that early (or even moderate) visual field defects often go unnoticed, particularly if only one eye is affected.

Useful questions to ask are:

- Have you noticed if any part of your vision is missing in either eye? Have you noticed any gaps in your vision?
- If you close each eye in turn, does what you see differ from one eye to the other?

In addition, it is essential to enquire about past ophthalmic and medical history, concentrating on family history, dietary/ drug/smoking/alcohol history, and whether there are any additional ophthalmic or neurological symptoms.

Visual field testing using confrontation only takes a few minutes, and should be mastered by all eye care workers, since it can provide a lot of useful information. Initially it is best to try and detect gross (absolute) defects. Position yourself in front of the patient, facing her/him with your face level with that of her/his, at a distance of about a metre. A comparison of examiner and patient fields is made, the assumption being that you, as the examiner, have normal visual fields (this is another reason why you should undergo visual field testing yourself).

First test the binocular visual field and then test each eye separately. A defect is detected by the absence of a patient response to the showing of a target, when the target is visible to you.

### Testing to confrontation with both eyes open

1. Ask the patient to stare directly and steadily into your eyes. Staring can cause embarrassment or awkwardness, so allow the patient to rest and try again if they find it difficult to look at you so directly. Check that the patient can look steadily at your eyes while you look steadily at theirs. Ask the patient whether any part of your face is missing or indistinct.
2. Check the patient's left hemi-field by making a fist with your right hand and holding it in their left hemi-field, at eye level, just to the right of your face. Making sure that the patient is still holding your gaze, raise one to four fingers and ask how many fingers can be seen. To test the upper and lower quadrants, move your hand up and to the right, and down and to the right, repeating the test at various points. This simple finger-counting test is particularly useful for detecting visual field loss due to neurological problems (such as strokes), but is only useful for patients with glaucoma when the visual field loss is severe.
3. To test the patient's right hemi-field and upper and lower quadrants, repeat the finger-counting test using your left hand, starting just to the left of your face and moving up and left and then down and left.
4. A useful, additional test to perform in patients with a suspected homonymous hemianopsia (i.e. loss of either the right or left field of vision in both eyes, often from a stroke) is to test for sensory inattention. Hold both hands up and wiggle the fingers of the right hand, followed by those of the left hand in each hemi-field. If the patient sees the moving fingers, then wiggle one finger of each hand at the same time – if the patient can only see movement on one side then they may have a subtle hemianopia.

### Testing each eye to confrontation

**Figure 5:**
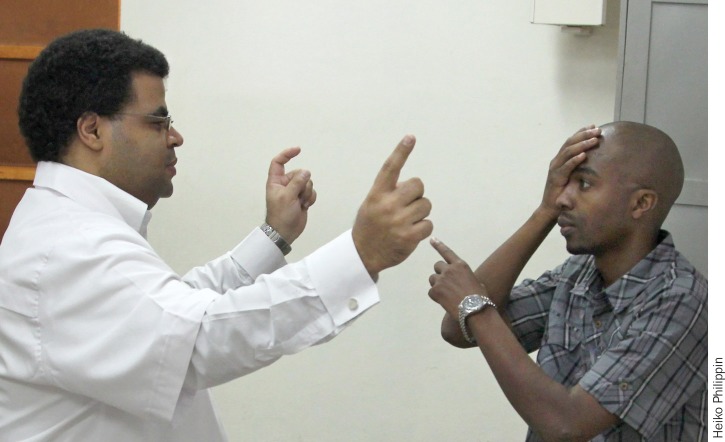
Testing visual fields to confrontation. The examiner's left eye is closed, so he can compare the field of his right eye with the field of the patient's left eye. TANZANIA

1. Ask the patient to cover their own eye with the palm of their hand (not their fingers, as it is easy to peep between fingers). Remember that you should close your eyes in turn too, so that you are comparing the field in your right eye with the field of the patient's left eye, for example (Figure 5).
2. Do the finger counting test first (static testing). Be sure to test on both the left and the right for each eye tested.
3. Next, bring your target finger from the far periphery in towards the central region (kinetic testing). Ask the patient to say when they first see the target. Repeat from several different directions, ensuring that the full 360° for each eye is tested. The examiner should remember to perform kinetic testing at a speed appropriate for the patient's responses.
4. Next, test the peripheral field with a white-headed neurological pin (beyond a central 30° radius) and the central field with a red-headed neurological pin (within a 30° radius). Testing with neurological pin targets gives much more accurate results than testing with fingers, and can detect earlier visual field loss. Red-headed neurological pins are also useful for assessing the size of the blind spot (e.g., with papilloedema), again by comparing the size of your blind spot with that of the patient's. In addition, red-headed neurological pins can be used to test for red-desaturation in early optic nerve disease.

## Part 4 Other visual field tests

This article does not cover all the details relating to the use of automated devices and users fortunate to have access to such devices should read and carefully adhere to the manufacturer's instruction manuals. Learning how to fully interpret standard automated perimetry (SAP) visual field printouts is also beyond the scope of this article and the reader is advised to source this information from an appropriate textbook. Readers with internet access can visit the Community Eye Health Journal website (www.cehjournal.org) for an additional online-only article about glaucoma and visual fields.

### Amsler chart testing

**Figure 6:**
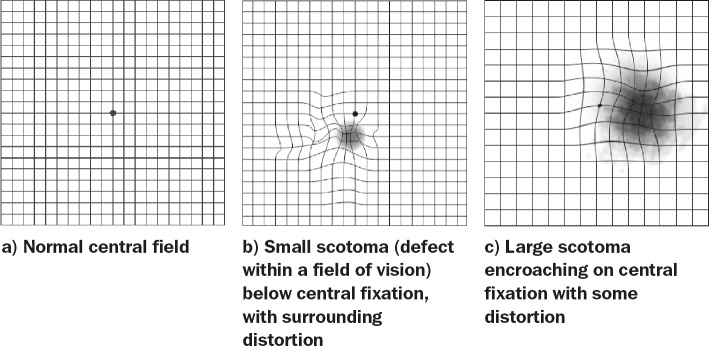
Amsler grid, when viewed by someone with normal central vision (a) and by people with a problem with their central visual field (b and c)

A printed grid (Amsler chart) can be used to detect subtle central defects (uncommon in patients with glaucoma) as well as paracentral defects (fairly common in patients with glaucoma -especially those with normal tension glaucoma). Test one eye at a time, correcting for any near refractive errors. Patients should hold the chart at a comfortable reading distance from their uncovered eye, and stare at the central spot of the grid. Ask them to identify and then point out any areas where the grid is missing or distorted. Missing areas may suggest paracentral glaucomatous visual field loss, whereas distortion is more common with macular disorders (Figure 6).

### Tangent screen testing (campimetry)

**Figure 7:**
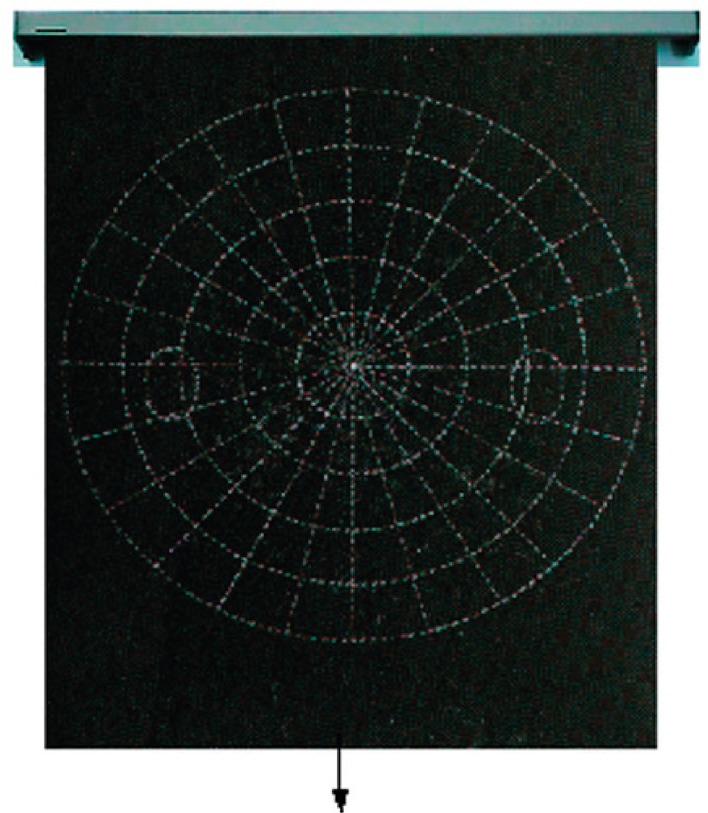
Bjerrum tangent screen. Tangent screens are useful for testing the central visual field, up to 30°

Tangent screen testing uses a flat testing surface and is useful for testing the central visual field, but for testing beyond 30° its value is limited (Figure 7). The tangent screen is usually a black felt screen mounted on a wall and testing is performed while the patient is sitting down. The screen should be well illuminated and appropriate for the specific type of test and target used. Most screens have circular white stitching or markings every 5° from a central fixation spot, up to 30°. The screen also has radial markings around the fixation point that start at the 180° meridian and are usually spaced 22.5° apart.

A disadvantage of campimetry is that no permanent record is generated, however, it can provide a convenient and quick way to obtain an impression of a field defect in a more formal manner than is achieved by confrontation testing.

### Goldmann perimetry

**Figure 8:**
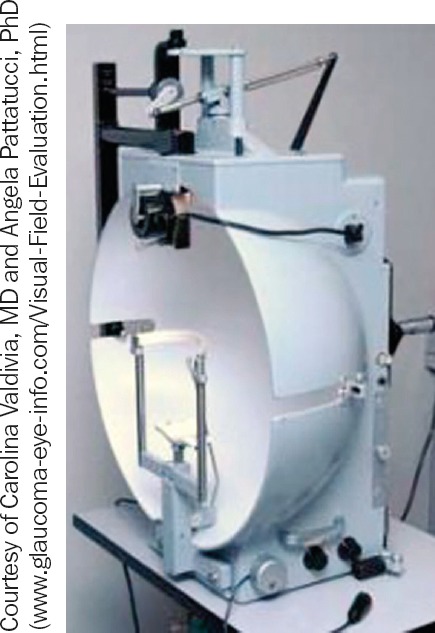
Goldmann perimeter

The Goldmann visual field analyser or perimeter (Figure 8) is a device that enables kinetic visual field testing, is more standardised than tangent screen testing campimetry), and generates a permanent record of the visual field, making it more sensitive, reproducible, and better for detecting change overtime. Many of the practical principles of Goldmann perimetry are similar to those relating to campimetry (and are not, therefore, repeated in this section).

The Goldmann perimeter consists of an illuminated hemispheric bowl upon which target spots of light are shone and moved from non-seeing regions to seeing regions. For kinetic testing, the examiner is able to move the target where they choose throughout a test, observe that the patient's eye is fixating on the fixation spot, communicate with the patient and document boundaries (isopters) between seeing and non-seeing regions to produce an exact drawing of visual fields. For static testing, the test target can be projected statically at a single location and the brightness increased until the patient responds that the target has been seen. The Goldmann device allows control of the luminance of both the background and stimulus targets and it is important that the device is calibrated on a regular basis to ensure that individual visual field plots can be compared with others; see the device calibration instructions. Target size is classified by Roman numerals (I–V), gross intensity of the target by Arabic numerals (1–4) and fine intensity by letters (a–e). Many different target stimuli can be selected (varying in size and luminance) and the III4e target with a diameter of 0.05° and area of 4 mm2 has subsequently become a standard target for many types of automated perimeter (see below).

### Standard automated perimetry

Automated visual field analysers have been developed, but these are expensive and not yet available in all departments throughout the world. However, research has shown that glaucomatous visual field loss is best detected and is managed with high reliability when automated perimetry is performed. SAP machines are highly technical and use intelligent computer software. It is hoped, therefore, that SAP will be used more widely with time.

SAP testing may be performed as a threshold or suprathreshold analysis. With suprathreshold analysis, the intensity of the stimulus target is not reduced to the level of detection/non-detection. A threshold analysis is more sensitive, but takes longer and is more susceptible to detecting artefacts (changes in the data that are due to the testing process itself, and not as a result of actual problems with the visual field).

SAP analysis provides the following:

- reliability indices (test duration, fixation losses, false-positive and false-negative error scores)
- a pictorial grey-scale plot of the visual field
- a plot of raw data sensitivities for each test spot
- global indices (in dB) indicating how the height and shape of the patient's hill of vision deviates from normal. Mean deviation gives the average difference between the patient's overall visual field sensitivity compared to a normal, age-corrected, reference field. Pattern standard deviation gives the standard deviation of the tested spot deviations from normal, thereby providing a measure of the degree to which the shape of a patient's field differs from normal
- a total deviation plot, together with a probability map indicating the likelihood for each missed point that it is abnormal
- a pattern standard deviation plot, together with its probability map
- analyses of change in visual field sensitivity with time
- results of the glaucoma hemi-field test – a relatively crude test for detecting glaucoma, based on the assumption that glaucoma frequently affects the superior hemi-field more than the inferior hemi-field or vice versa.

The key feature of a glaucomatous visual field defect is an abnormality on the pattern standard deviation plot, which also shows on the total deviation plot.

A field defect on the total deviation plot, in the absence of a defect on the pattern standard deviation plot, can be due to glaucoma (diffuse field loss), but is more likely to be due to media opacity (e.g. cataract).

A field defect that appears more extensive on the total deviation plot than on the pattern standard deviation plot may indicate co-morbidity (e.g. cataract and glaucoma).

**Figure 9:**
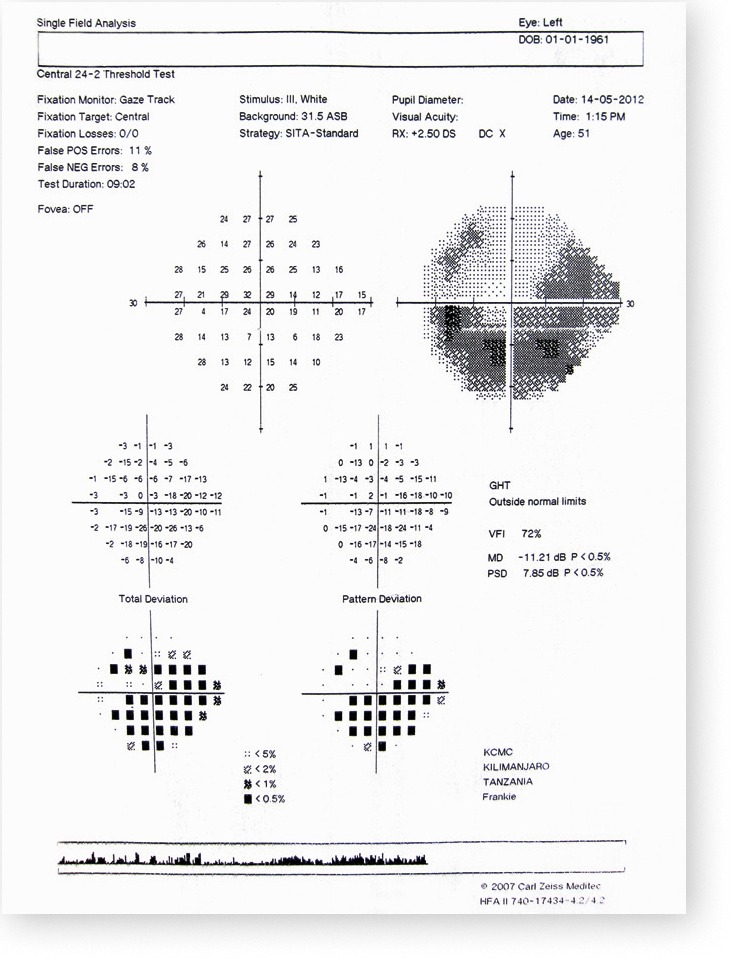
A standard automated perimetry printout for someone with advanced primary open-angle glaucoma

